# Supraspinatus tendon repair using anchors: a biomechanical evaluation in the rabbit

**DOI:** 10.1186/s13018-018-0773-6

**Published:** 2018-03-27

**Authors:** Hakim Louati, Hans K. Uhthoff, Kathryn Culliton, Odette Laneuville, Peter Lapner, Guy Trudel

**Affiliations:** 1 Bone and Joint Research Laboratory, University of Ottawa, Ottawa, Canada; 2 Bone and Joint Research Laboratory and Division of Orthopaedic Surgery, Ottawa Hospital Research Institute, University of Ottawa, Ottawa, Canada; 3 Orthopaedic Biomechanics Laboratory, Division of Orthopaedic Surgery, University of Ottawa, Ottawa, Canada; 4 Department of Biology, University of Ottawa, Ottawa, Canada; 5 Division of Orthopaedic Surgery, Ottawa Hospital Research Institute, University of Ottawa, Ottawa, Canada; 6 Bone and Joint Research Laboratory, Department of Medicine, Division of Physical Medicine and Rehabilitation, University of Ottawa, Ottawa, Canada; 7 Division of Physical Medicine and Rehabilitation, The Ottawa Hospital Rehabilitation Centre, 505 Smyth Rd., Ottawa, ON K1H 8M2 Canada

**Keywords:** Rotator cuff, Biomechanical, Channeling, Supraspinatus tendon, Rabbit, Decortication

## Abstract

**Background:**

Arthroscopic rotator cuff repairs are mostly secured with suture anchors and often supplemented by footprint decortication. The objectives of this study were to characterize the strength of bone–tendon healing following anchor repair and assess the effect of channeling the supraspinatus (SSP) humeral footprint 1 week ahead of reattachment surgery.

**Methods:**

One hundred twelve rabbits underwent unilateral detachment of one SSP tendon and were randomly assigned to two groups: channeling the footprint at time of detachment and no channeling. One week later, reattachment was performed using an anchor. The repaired and contralateral shoulders were harvested at 0, 1, 2, or 4 weeks after repair and mechanically tested to failure. Outcome measures included load at failure, stiffness, and site of failure.

**Results:**

Anchor fixation had a mean load at failure of 81 ± 32 N and a stiffness of 27 ± 9 N/mm immediately after repair compared to 166 ± 47 N and 66 ± 13 N/mm in the contralateral (both *p* < 0.05). Mechanical recovery of the reattached SSP tendon was achieved after 4 weeks (221 ± 73 N, 206 ± 59 N, and 198 ± 49 N in the channeling, no channeling, and contralateral groups, respectively, *p* > 0.05). The dominant site of failure shifted from the footprint at 0/1 week to bone avulsion/mid-substance tear at 4 weeks (*p* < 0.05). There were no differences in outcomes between the channeling and no channeling groups.

**Conclusions:**

This study is the first of its kind to provide quantitative data on the mechanical properties of the enthesis following anchor repair in a rabbit model. Anchor repair led to rapid and complete restoration of SSP mechanical properties. Further evidence is needed before recommending channeling ahead of repair surgery.

## Background

Arthroscopic repair of rotator cuff tears using anchor fixation has decreased the invasiveness of surgery allowing for faster recovery and more favorable clinical outcomes [1–3]. However, reformation of the enthesis (the tendon–bone transition zone at the footprint), a condition necessary for a long-lasting anatomical outcome, is often not achieved [4]. Published postoperative bone–tendon defect rates following arthroscopic repair are as high as 86% in tears larger than 3 cm and 88% in tears smaller than 3 cm [5, 6]. The loss of anatomical continuity at follow-up examination is foremost due to tendon dehiscence or re-tear [7–9]. The reconstructed supraspinatus (SSP) is mechanically weakest immediately following repair [10]. Therefore, establishing and maintaining tendon–bone continuity during this period is critical to successful outcomes [11]. Consequently, interventions to improve enthesis reformation at the SSP footprint in the immediate postoperative period could lower tendon dehiscence rates [10].

To improve the initial strength and anatomic reformation of the enthesis, material and technical innovations including various anchor fixations and suture configurations such as single- and double-row repair and suture bridging are used [12, 13]. Biological augmentations such as scaffolds, platelet-rich plasma, stem cell transplants, growth factors, and footprint decortication have also been investigated [7]. Creating communication channels between the footprint and the deep “red” or hemopoietic bone marrow seems a promising strategy for biological augmentation allowing more pluripotential cells of the bone marrow to contribute to healing of the reattached tendon [14]. These deep bone marrow communication procedures have shown favorable clinical outcomes [15, 16]. Jo et al. drilled channels at the SSP tendon footprint at the time of repair surgery in 57 patients and compared them with 67 control patients [17]. After 2 years, the channeled group re-tear rate (22%) was half that of the controls (45%). Kida et al. attributed the improvement to the presence of bone marrow-derived mesenchymal stem cells (MSCs) at the time of repair leading to faster and better enthesis reformation [6]. Channeled repairs showed higher loads at failure compared to no channeling. However, opening deep bone marrow communication at the time of surgery may not confer immediate benefits since recruitment and activation of pluripotent cells from the bone marrow to the surgical site takes time. In one study, bone marrow-recruited cells were still undifferentiated at week 2, differentiating into fibroblast-like cells by 4 and 8 weeks [17]. In a second study, differentiation from precursor cells to fibroblast-like cells happened over 4 weeks [18].

Building on these investigations, we postulated that deep channeling of the footprint prior to reattachment surgery could biologically prime the repair site. Bone marrow pluripotential cells would be recruited before repair surgery, migrate to the footprint, divide, and become activated. Then, upon surgical supraspinatus reattachment, larger numbers of undifferentiated cells would immediately be available on-site to initiate enthesis reformation and could shorten or eliminate the recruitment time associated with channeling. Critically, the time before biological healing sets in coincides with the mechanically weakest moment of reattachment surgery, where it is approximately 20% of normal [11]. Therefore, the time interval before biological healing starts bears the highest risk for tendon dehiscence from the bone, eventually leading to surgical failure. Channeling prior to repair could favor a more successful initial repair, as suggested in earlier trials, and may result in better biomechanical properties in the first 4 postoperative weeks [14, 15, 17].

The development and testing of animal models of rotator cuff tears and repair is crucial to providing evidence-based care as they allow for controlled experimentation without confounding factors that limit clinical studies [11]. Arthroscopic repair of rotator cuff tendons is widespread clinically and has become the standard of surgical care. Yet, while animal models have tested primary anchor fixation, fewer studies have studied the healing after anchor repair in longitudinal animal models [19–21]. There is a need for preclinical models that better simulate the standard of surgical care, and establishing a baseline for comparison of associated mechanical parameters is critical to the investigation of novel techniques and theories aimed at improving outcomes.

The study objectives were (1) to characterize the SSP tendon–bone healing mechanically in the first 4 postoperative weeks after anchor fixation in a rabbit model of SSP repair and (2) to study the effect of channeling of the SSP humeral footprint performed 1 week prior to reattachment. We hypothesized that (1) SSP repair using an anchor fixation in a rabbit model will restore load at failure, stiffness, and mode of failure to match those of the contralateral shoulders by 4 postoperative weeks and (2) channeling the humeral footprint 1 week prior to surgical reattachment will accelerate the restoration of biomechanical properties.

## Methods

### Animals

**Fig. 1:**
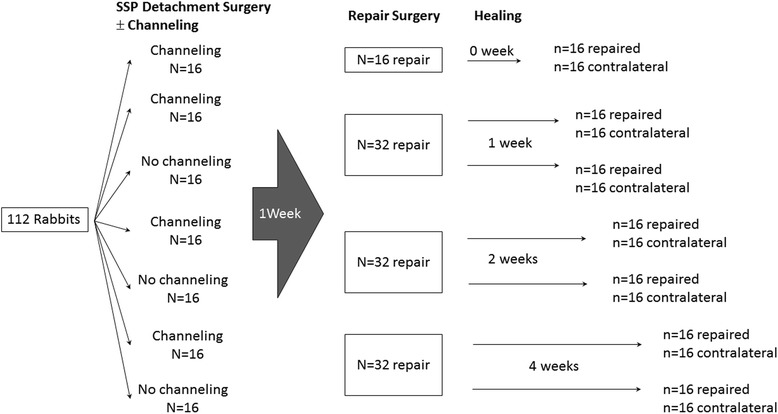
Study protocol

One hundred twelve adult female New Zealand white rabbits weighing 3.0 ± 0.3 kg were used. The experimental procedures were approved by the Institutional Animal Care Committee and Research Ethics Board (Protocol ME-2479). All rabbits had one shoulder randomly selected to undergo a two-time surgery, first a detachment of the SSP tendon, followed by reattachment surgery 1 week later. During detachment surgery, the footprint of half of the rabbits underwent channeling (channeling group) and the footprint of the remaining rabbits was untouched (no channeling group). The 7-day delay between channeling and repair was selected (1) based on preliminary histological data on rabbit footprint channeling, wherein 7 days after channeling corresponded to the greatest subenthesial cellular proliferation, and (2) to replicate an ongoing clinical trial investigating channeling 7 days prior to repair (CTI: NCT01706978, clinicaltrials.gov). One hundred twelve contralateral shoulders served as controls. All rabbits were housed individually at 22 °C on a 12-h light/dark cycle with access to water and standard chow and allowed weight bearing after surgery. Rabbits were euthanized at 0, 1, 2, or 4 weeks following reattachment surgery in groups of *n* = 16 (Fig. 1). The 4-week maximum postoperative follow-up was based on a previous study showing complete restoration of rabbit SSP mechanical strength and stiffness between weeks 2 and 6 after repair [10]. Sample size was calculated based on previous experiments: detection of a difference in the primary outcome measure (load at failure) of 33% with a power = 0.80 and alpha = 0.05 required 15 rabbits per group [10]. Allowing *n* = 1 for technical failures resulted in a sample size of *n* = 16.

### Surgical methods

Detachment surgery consisted of sharply detaching the SSP insertion from the greater tuberosity, simulating a complete tear, as detailed in a previous investigation [22]. The distal, free end of the tendon was wrapped in a polyvinylidene membrane (5 μm, Durapore; Millipore, Bedford, MA) to prevent spontaneous reattachment. Following detachment, half of the rabbits underwent bone channeling. Channeling involved partitioning the SSP humeral footprint into four quadrants and drilling a 1-mm-diameter hole at the center of each quadrant. The holes were drilled to a depth of approximately 10 mm to ensure deep communication with the red bone marrow of the epiphysis. The other half of the operated animals was not subjected to channeling. The deltoid was then closed followed by skin closure. The rabbits received fentanyl and buprenorphine for 3 days postoperatively and were allowed to roam freely in cages with unlimited access to food and water.

Reattachment surgery was performed 1 week later and was the same for the channeling and no channeling groups. The incision was reopened. A curette was used to clear any tissue or fluid that had accumulated at the drill sites and shallowly decorticate the footprint. The free distal SSP tendon stump was mobilized and the Millipore wrapping removed. A single 3-mm Bio-FASTak® anchor with #2 FiberWire sutures (Arthrex, Naples, FL) was inserted lateral and distal to the footprint in the cortical bone, and the tendon was reapproximated to the footprint using a horizontal mattress stitch as described by Boileau et al. [23, 24]. The wound was closed as after the first surgery.

### Collection of specimens

All rabbits were euthanized with a pentobarbital overdose at the specified times. Both the operated and contralateral shoulders were harvested from each animal and individually wrapped in saline-soaked gauze to avoid dehydration. The scapula, rotator cuff muscles, and proximal humerus were harvested en bloc and immediately frozen at − 20 °C.

### Biomechanical testing

The specimens were thawed gradually to room temperature. The SSP and proximal humerus were isolated to ensure that only the SSP attached to the humeral head contributed to the mechanical evaluation. The humerus was fixed in a bone clamp which compressed the humeral head to prevent premature tensile failure of the bone. The bone clamp was then potted in an adaptor cup using a low-melting bismuth alloy. The SSP muscle was clamped in a cryogenic fixation unit (CFU) that uniformly transfers load to the tendon by external freezing of the muscle using liquid nitrogen of the myotendinous junction embedded in a saline solution [25]. The humeral head to myotendinous junction test length was standardized to 24 mm.

**Fig. 2:**
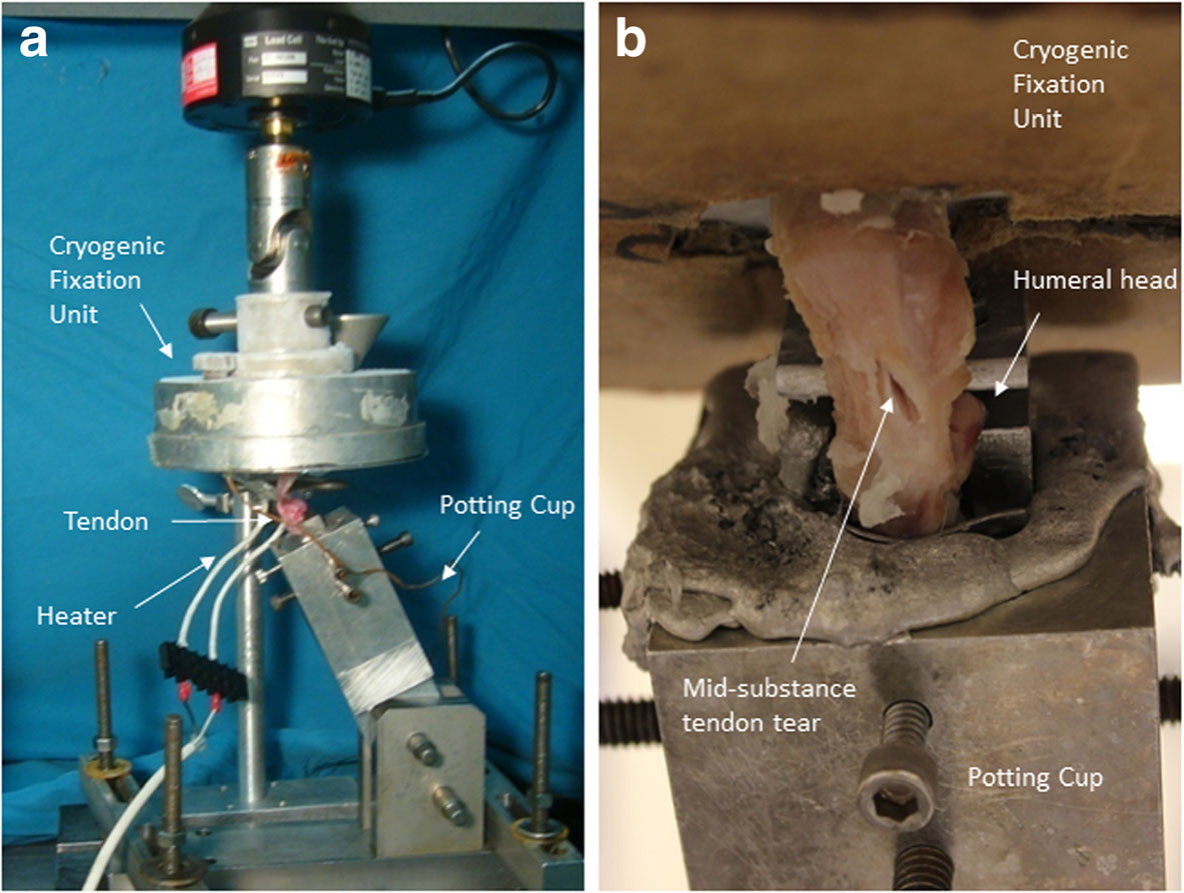
**a** Mechanical testing with the cryogenic fixation unit. **b** A close-up of specimen that failed from a mid-substance tendon tear. Note the typical composite material failure mode with rupture and delamination of tendon fibers

Both fixtures were mounted on an electro-mechanical load frame with a 2.5-kN load cell (MTS Sintech 1G; MTS Systems Corporation, Eden Prairie, MN, USA) with the CFU attached to the crosshead and the bone clamp attached to the base (Fig. 2). Petroleum jelly was applied to the exposed tendon to prevent drying, and a heater surrounded the tendon to ensure that the enthesis and tendon test length remained at room temperature. The tendons were positioned along their anatomic direction of pull, at an angle of 45° to the longitudinal axis of the humeral shaft. The specimens were preconditioned for 12 cycles from a preload of 5 N to a peak load of 50 N at a loading rate of 15 N/s. This was followed by a tensile load to failure at a rate of 1 mm/s where a 50% drop in tensile strength was defined as the breaking point. The load and displacement data were collected, and the mode of failure was noted. The load at failure was determined using TestWorks 4 software (MTS Systems Corporation, Eden Prairie, MN, USA). Stiffness was calculated by fitting a regression line to the linear portion of the load–displacement curve for each specimen. We defined three sites of failure: footprint failure, bone avulsion, and mid-substance tendon tear (Fig. 2).

### Data and statistical analysis

Data are expressed as mean ± 1 standard error of the mean (SEM). Statistical analysis was performed with SPSS (v 17, IBM, New York, USA). Load at failure and stiffness over duration of healing (0, 1, 2, or 4 weeks) and across the three shoulder groups (channeling, no channeling, and intact contralateral) were compared using a two-way ANOVA followed by Bonferroni–Holm post hoc tests for pairwise comparisons. The association between site of failure and (1) intervention, (2) duration of healing, and (3) load at failure were tested using a Kruskal–Wallis test, followed by post hoc Mann–Whitney tests for pairwise comparisons. A *p* value of 0.05 or less was considered statistically significant.

## Results

**Fig. 3:**
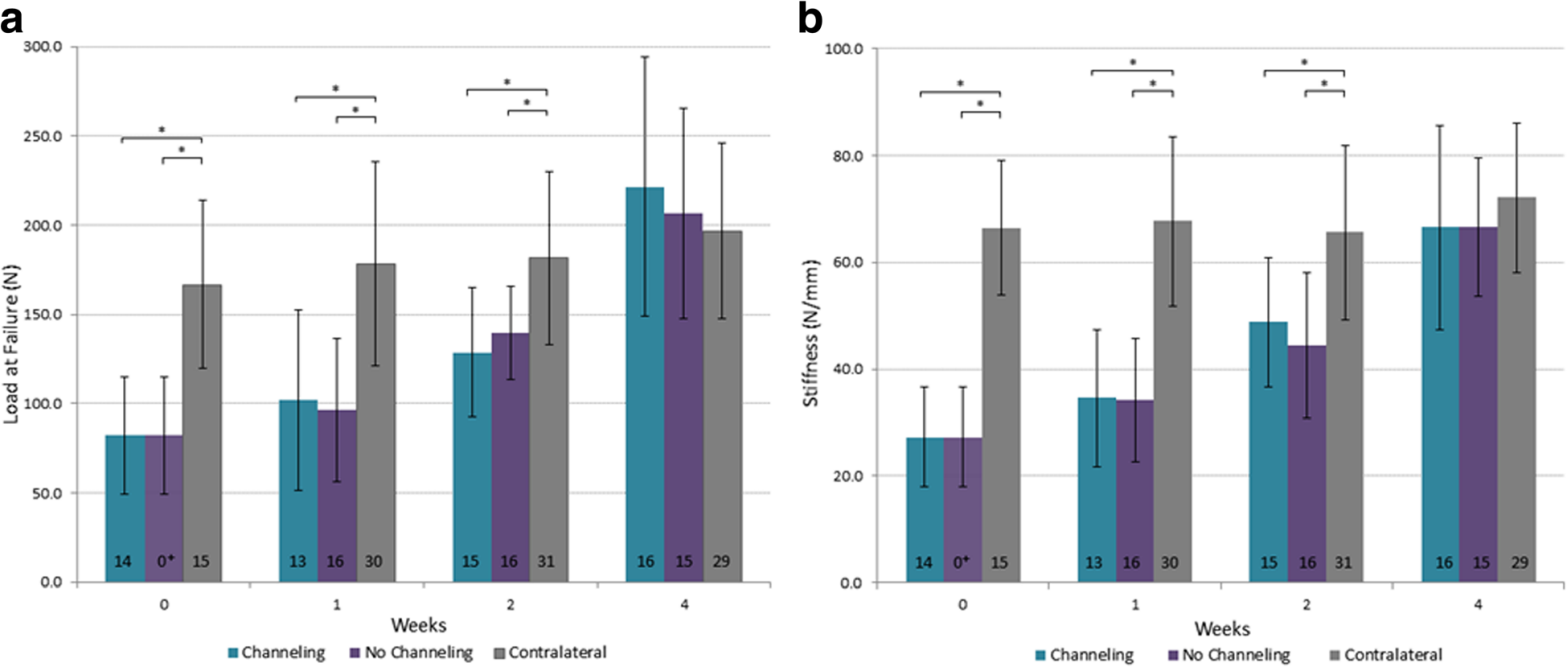
Mean ± 1SD load at failure (**a**) and stiffness (**b**) of the channeling, no channeling, and contralateral rabbit supraspinatus tendons following anchor repair (0 to 4 weeks). *p < 0.05; plus sign indicates that at week 0 (no enthesis reformation), no channeling data assumed identical to channeling data

**Fig. 4:**
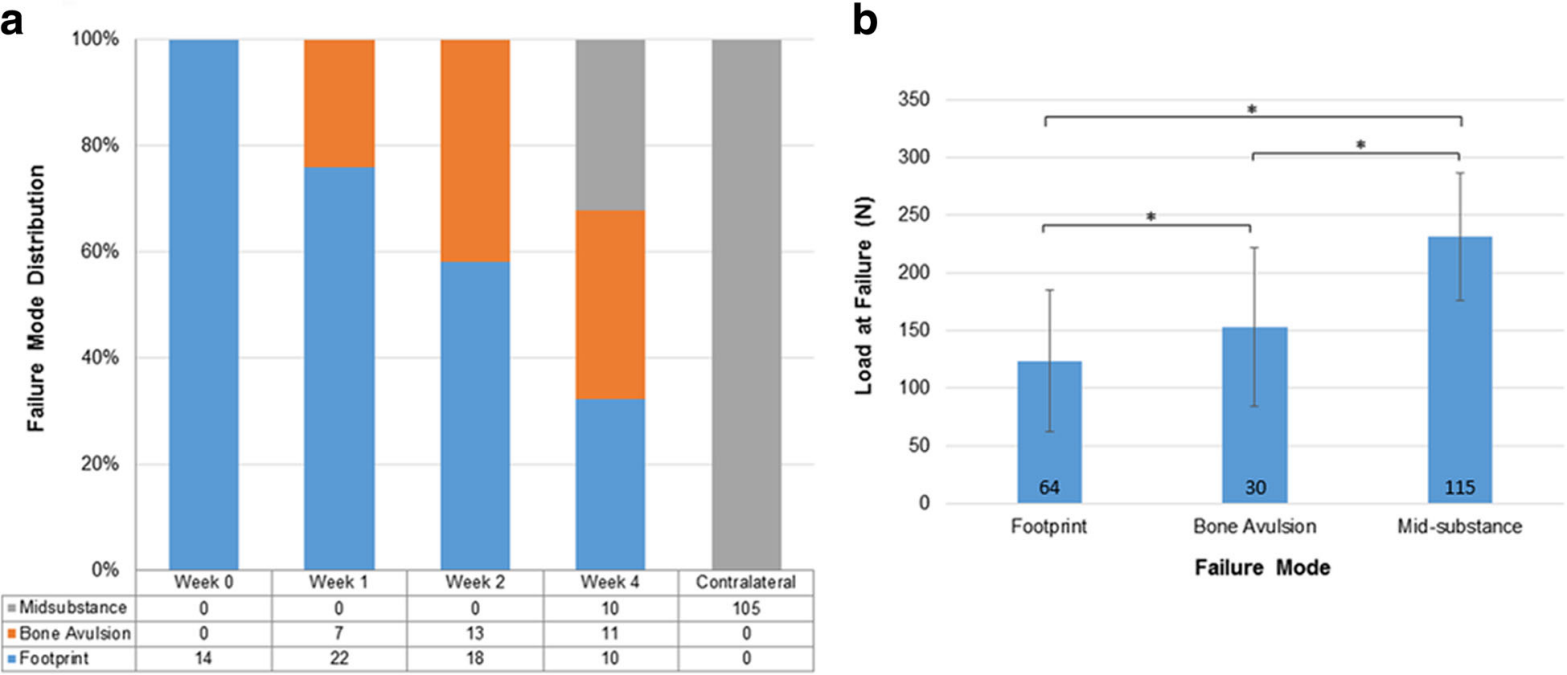
Mode of failure of the anchor-repaired and contralateral SSP (0 to 4 weeks). **a** Distribution data. **b** Mean ± 1SD load at failure by mode of failure. Error bars = 1SD; *p < 0.05. The number of specimens is indicated on the bar

Both shoulders of 112 rabbits (224 shoulders) were harvested. Fourteen shoulders were excluded from the mechanical data analysis due to damage during surgery (*n* = 4), dissection (*n* = 5), or mechanical testing preparation (*n* = 5). Final sample sizes are shown in Figs. 3 and 4.

### Load at failure and stiffness

Load at failure increased significantly with increased durations of healing (0, 1, 2, or 4 weeks; *F*(3,198) = 32.1, *p* < 0.001; Fig. 3a). Load at failure was also significantly different between the three shoulder groups (channeling, no channeling, contralateral; *F*(2,198) = 21.3, *p* < 0.001; Fig. 3a). Finally, there was a significant interaction between shoulder groups and duration of healing (*F*(5,198) = 7.22, *p* < 0.001). Post hoc testing revealed that loads at failure were significantly lower in repaired shoulder groups (both channeling and no channeling) than in the contralateral shoulders at weeks 0, 1, and 2 (all *p* < 0.01; Fig. 3a). Both repaired shoulder groups had reached contralateral loads at failure by week 4 (*p* > 0.05). There was no significant difference in load at failure with channeling compared to no channeling at any time point (all *p* > 0.05; Fig. 3a).

Similarly, stiffness increased significantly with increased durations of healing (*F*(3,198) = 29.4, *p* < 0.001; Fig. 3b) and was significantly different between the shoulder groups (*F*(2,198) = 65.9, *p* < 0.001; Fig. 3b). There was a significant interaction between shoulder groups and duration of healing (*F*(5,198) = 7.96, *p* < 0.001). Stiffness was significantly lower in both repaired shoulder groups than in the contralateral at weeks 0, 1, and 2 (*p* < 0.01; Fig. 3b). Both repaired shoulder groups had reached contralateral stiffness by week 4 (*p* > 0.05). There was no significant difference in stiffness with channeling compared to no channeling at any time point (all *p* > 0.05; Fig. 3b).

### Sites of failure

All contralateral shoulders across all time points failed through mid-substance tendon tear near the myotendinous junction (Fig. 4). The site of failure for the two repair shoulder groups combined, varied with duration of healing (*p* < 0.001). At week 0, all repaired tendons failed at the footprint due to suture pullout. The sites of failure shifted from a mix of a rupture at the footprint and bone avulsion at 1 and 2 weeks toward bone avulsion and mid-substance failures by 4 weeks (*p* < 0.05; Fig. 4). Failure at the footprint was associated with the lowest load at failure, bone avulsions were associated with higher loads at failure, and mid-substance tears were associated with the highest loads at failure (*p* < 0.05; Fig. 4). There were no statistically significant differences in site of failure between channeling and no channeling groups across all time points (*p* > 0.05).

## Discussion

The novel evidence contributed by this study lies in the positive effect of anchor fixation. The strength of fixation with a suture anchor compared favorably with the literature using the transosseous cuff repair technique [8]. Transosseous fixation also led to similar strength between the repaired and contralateral groups at 4 weeks post repair [8]. This is important as transosseous repair, while recently regaining popularity in arthroscopic applications, has largely been replaced by anchor fixation even though the strength of anchor fixation during the early phases of healing and enthesis reformation had never been tested experimentally [26].

Data obtained immediately after single-anchor SSP repair resulted in a load at failure of 82 N or 49% of the load at failure of the intact contralateral shoulder (Fig. 3). This is attributed to the mechanical strength of the sutures of the anchor in the absence of enthesis reformation. Four weeks after anchor fixation, the load at failure and stiffness progressed to those of the contralateral shoulder confirming our first hypothesis. The anchor fixation experimental data obtained in this study constitutes evidence supporting the use of the anchor technique.

To our knowledge, only one other study used a rabbit SSP anchor repair model. Ozbaydar et al. reported failure loads in the range of 5–9 N [9]. These values are an order of magnitude below those reported in the current study. This may be due to experimental differences in the testing setup since the current study relied on cryogenic fixation to ensure a uniform soft tissue load distribution. Normal rabbit SSP failure loads have been reported in the range of 185–343 N [25, 27]. Contralateral failure loads in the current study ranged between 166 and 196 N and thus were in keeping with previous studies and validated the model and mechanical protocol. Stiffness is another indicator of repair integrity as it represents the structure’s resistance to deformation. The stiffness results in this study paralleled the load at failure data with an initial week 0 stiffness of 27 N/mm or 41% of the contralateral and progressive improvement in subsequent weeks to contralateral levels by week 4.

Site of failure analysis adds an important perspective to SSP healing after repair. Normal contralateral tendons failed at the tendon mid-substance, near the myotendinous junction. The site of failure of repaired tendons evolved with the postoperative duration. Immediately postoperatively, all the repaired tendons failed at the footprint, with the sutures cutting through the tendon as no enthesis reformation had occurred. Progressive enthesis reformation led to a stronger enthesis and shifted the mode of failure more proximally to the tendon proper (Fig. 4). Significantly, these results support the notion that early postoperative distal SSP tendon failures are due to tendon dehiscence while later failures are due to mid-substance tear or tendon re-tear. Taken together, the restored strength, stiffness, and mode of failure 4 weeks after single-anchor repair confirmed our first hypothesis. By using a repair technique that had quantifiably good initial strength and stiffness, good stability during the initial postoperative period, and good tendon–bone contact, and hence meeting the criteria for effective cuff repair, this study provides basic evidence supporting the clinical practice of using anchors for rotator cuff repair [26].

Footprint channeling 1 week prior to repair did not improve the mechanical properties of repaired SSP tendons compared to no channeling, contrary to our second hypothesis. At least four factors may explain the negative results: (1) cleaning of the footprint co-intervention at the time of repair, (2) failure of pluripotential cells reaching the site repair, (3) timing of the channeling intervention, and (4) the extent of channeling. Current clinical practice includes decortication of the SSP humeral footprint to induce superficial communication with the bone marrow at the time of repair. Decortication has shown improvements in surgical outcomes similar to channeling by enabling the migration of cells and mediators from the subenthesial adipose bone marrow [15]. In the current study, the cleaning of the footprint co-intervention performed for both the channeling and no channeling shoulders could have masked the effect of channeling. Thus, the cleaning of the footprint could have had a greater effect on healing and may have had a greater effect on the recruitment of activated pluripotential cells from the hemopoietic marrow to the repair site than bone channeling. A study in rats showed that preserving fibrocartilage at the footprint in addition to channeling showed superior ultimate force to failure of transosseous repairs [28].

This study has limitations inherent to animal models. We sharply transected healthy SSP tendons and repaired them a week later. While this model simulates a traumatic tendon rupture and repair, it does not replicate the predominantly chronic degenerative cuff tears seen in clinical practice. As well, in the quadruped model, the shoulder is loaded as tolerated postoperatively, while in humans, the shoulder is protected following cuff repair. Both may lead to a different progression of healing and limit the generalization of the findings.

## Conclusion

This study is the first of its kind to provide longitudinal quantitative data on the mechanical properties of the healing enthesis and tendon following anchor repair in a rabbit model. These data can be used as a baseline for the assessment of endogenous and exogenous augments, materials, and techniques aimed at improving repair of the rotator cuff and tendon–muscle healing in general. Furthermore, pre-repair channeling had no significant effect on mechanical properties.

## Abbreviations

CFU: Cryogenic fixation unit
MSCs: Mesenchymal stem cells
SSP: Supraspinatus
